# European Cancer Organisation's Inequalities Network: Putting Cancer Inequalities on the European Policy Map

**DOI:** 10.1200/GO.22.00233

**Published:** 2022-10-17

**Authors:** Hendrik Van Poppel, Nicolò Matteo Luca Battisti, Mark Lawler, Teodora Kolarova, Jacqueline Daly, Katie Rizvi, Robert Greene, Guy Buyens, Kathy Oliver, Richard Price, Nela Osmanovic, Enea Venegoni

**Affiliations:** 1 European Association of Urology, Arnhem, the Netherlands; 2 Katholieke Universiteit Leuven, Leuven, Belgium; 3 International Society of Geriatric Oncology, Châtelaine, Switzerland; 4 The Royal Marsden NHS Foundation Trust, London, United Kingdom; 5 Queens University Belfast, Belfast, Northern Ireland; 6 International Neuroendocrine Cancer Alliance, Boston, MA; 7 East Galway & Midlands Cancer Support, Ballinasloe, Ireland; 8 Youth Cancer Europe, Cluj Napoca, Romania; 9 HungerNdThirst Foundation, Amsterdam, the Netherlands; 10 Anticancer Fund, Meise, Belgium; 11 International Brain Tumour Alliance, Tadworth Surrey, UK; 12 European Cancer Organisation, Brussels, Belgium

## Introduction

The noted economist Stiglitz^1^ commented that inequality is a choice. Inequality comes from the policy decisions systems make. The European Cancer Organisation (ECO) considers this dictum applies to the prevalence of inequalities in cancer care. In 2020, ECO members chose to take action on inequalities by establishing a Focused Topic Network dedicated to advocating on the topic.^2^

The Network is co-chaired by Nicolò Matteo Luca Battisti (International Society of Geriatric Oncology) and Hendrik Van Poppel (European Association of Urology). More than 30 organizations participate including 15 European and international level health care professional organizations, nine patient organizations, five charities and foundations, and other invited stakeholders.

## Putting Inequalities on the EU Cancer Agenda

The Network commenced in 2020, a pivotal year politically for cancer in the European Union (EU). After the 2019 European Parliament elections, the incoming President of the European Commission, Ursula von der Leyen, alongside the new EU Commissioner for Health and Food Safety, Stella Kyriakides, committed^3^ to creating an EU cancer plan and spent a year consulting on its content.

The ECO Inequalities Network commenced early advocacy on the Plan content, recommending the Plan:

(a) Addresses inequalities as a theme connecting all initiatives including in prevention, early detection, treatment, and follow-up.
(b) Has inequalities measurement as a critical component. What gets measured gets done.
(c) Looks beyond inequalities between countries and includes social disparity indicators.

## Measuring Inequalities

The Network then allied with other organizations to promote the concept of a European Cancer Dashboard for inclusion in Europe's Beating Cancer Plan (EBCP).^4^ Such a measurement tool would better identify, compare, and prioritize action on cancer inequalities.

Our Inequalities Network was, therefore, delighted when EBCP was published in February 2021 with a commitment to a new Cancer Inequalities Registry.^5^ Much of the core principles and concepts of this Inequalities Registry equate well with the European Cancer Dashboard proposal.

## Developing the EU Cancer Inequalities Registry

The Network is working with the key agencies creating the Inequalities Registry. The three organizations developing the Registry are the European Commission's Joint Research Center; the Organisation for Economic Co-operation and Development; and the International Agency for Research on Cancer. Each provides a separate role. The Joint Research Center provides a new visualized expression of key indicators collected by the European Cancer Information System. An early version of the registry is now available.^2^ Indicators include cancer mortality; smoking, obesity, and physical activity rates; screening rates; and availability of different treatment modalities such as radiation therapy.

Meanwhile, the Organisation for Economic Co-operation and Development will publish country profiles with findings on cancer inequalities within and between the EU's 27 member states. International Agency for Research on Cancer is tasked with providing a comprehensive understanding of the inequalities in cancer care that exist within countries with an emphasis on social disparities.

## Our Areas of Ongoing Focus: Geographical and Social Disparities

### Ageism.

Cancer is mostly a disease that affects people later in life, with approximately 54% of new diagnoses and 70% of related deaths occurring in individuals aged 65 years or older in the United States.^6^ Older adults also have nearly 10 times the risk of developing cancer compared with younger counterparts.^7^ Nonetheless, older adults remain under-represented in oncology clinical trials.^8^ Moreover, older individuals with cancer are heterogeneous, and chronological age alone provides little information regarding tolerance and benefits of anticancer treatments in this population.^9^ A significant degree of variation in survival outcomes for individuals with cancer has been observed in the older age group among different countries.^10,11^ For example, in the EUROCARE-5 analysis, a gap of > 10% in age-specific relative survival at 5 years has been documented in adults diagnosed with cancer 75 years or older between the United Kingdom and Ireland and Southern Europe.^11^ Importantly, treatment variation for older adults with cancer has also been documented within and among different countries.^12^ Finally, geriatric assessments and driven multidisciplinary interventions should be a standard of care for older patients with cancer.^13-15^ To tackle these issues, multidisciplinary clinical initiatives on the basis of sound research data and educational activities for cancer workforce are essential for the advancement of the care of older adults with cancer globally.^16^ The network also has concerns around disparities related to age when it comes to inclusion criteria in clinical trials. For example, because of the 18-year-old limit to access clinical trial, adolescents and young adults end up not having access not only to innovative clinical trials but also to new therapies.^17^

### Sex.

ECO seeks systems of cancer care that are conscious of sex-related disparities in cancer care but also provide services in a sex-conscious manner. In their review on sex-related differences in colorectal cancer incidence, White et al^18^ (2018) showed that although minimal sex differences in survival by stage at diagnosis were detected, differences can be found in the pathway leading to diagnosis. Men have overall lower bowel screening uptake, whereas a higher proportion of colorectal cancer cases in women are diagnosed via emergency presentation. This not only shows differences in sex when approaching cancer health system but highlights the importance of considering external factors that might influence screening uptake at individual level and of including them in personalized treatment. It is worthy to mention that media coverage of cancer in men has been showed to be lower compared with cancer in women,^19^ which might influence awareness of screening programs and, consequently, willingness to screening uptake by men. In fact, disparities in awareness of cancer risks might also account for less screening uptake. In a sample of 3,608 participants who received questions about human papillomavirus (HPV), 78% of them knew HPV provokes cervical cancer, whereas only 29% provokes penile cancer and only 26% anal cancer.^20^ Moreover, men were less likely to have heard about HPV and HPV vaccine.^20^

Sex differences are detected in exposure to carcinogens at work. Scarselli et al^21^ evaluated exposure to 40 carcinogens among Italian workforce, concluding that women were most likely to be exposed to higher levels of carcinogens. However, a study investigating carcinogens exposure among the workforce in Australia showed that men workers were more exposed.^22^ This suggests need for further investigation in the factors behind carcinogens exposure at work that might account for sex differences.

### Ethnicity.

Disparities on the basis of race and ethnicity in cancer research and care arise both from the sociopolitical historical context and from systematic issues within the health system^23^ and have an impact at research, screening, and prevention levels.^22^ A retrospective study that included 23,123 women who received a diagnosis of nonmetastatic triple-negative breast cancer in the United States showed that non-Hispanic African American women received diagnosis at more advanced stages and were more likely to receive chemotherapy and surgery after diagnosis compared with White women.^24^ Shavers and Brown^25^ found that racial and ethnic disparities accounted for disparities within cancer-directed therapy, radiation therapy after breast-conserving surgery, clinical staging, and adjuvant surgery. These differences were not explained by differences accountable to clinical profiles, which suggest impact of nonclinical factors. Although European research on the levels of inequalities in cancer care related to racial and ethnic factors lags behind the United States, the lessons being learned across the Atlantic have potential application for the European Union. Entities working in cancer care should encourage and advocate for the collection of new data and improved research methodology to fully quantify the impact of factors accountable for disparities.

### Sexual orientation and gender identity.

Research on cancer care among LGBTIQ+ patients is still insufficient. Indeed, it is often the case that research on the LGBTIQ+ community lumps together the different sexual orientations and gender identities and, therefore, fails to address specific factors affecting the different groups within the community. For example, a recent study that included 600 transgender patients with cancer in the United States showed that even after correction for health insurance and exclusion of people who refused treatment, transgender patients tended to receive a diagnosis for lung cancer at a later stage and to have worse survival rates compared with cisgender people after a diagnosis for non-Hodgkin lymphoma, prostate cancer, and urinary bladder cancer.^26^ This could be linked to the disparities faced at the stage of screening, whereby screening among transgender individuals is lower than cisgender people,^25^ because of clinicians' unpreparedness on transgender bodies and to bureaucratical impediments, for example, not receiving screening reminders after rectifying sex on legal documents. Additionally, LGB cancer survivors experience higher rates of depression and difficulties related to relationship.^27^ The data highlight a need for conducting further research in this topic and to address the need for stratifying research for different communities and to implement personalized medicine.

### Geographical disparities.

The gap between Central East Europe (CEE) and West Europe has been pointed out several times.^28^ For example, it is evident from the Time to Act COVID-19 and Cancer Data Navigator that there is a difference in the data submitted for West Europe compared with CEE, whereby for the latter area fewer data points are available.^29^ This could be indicative of a lower amount of relevant research and reporting being conducted in Eastern countries. When looking at the European Cancer Inequality Registry, it appears that for many of the indicators compared by country, such as cancer mortality, data for Eastern countries are worse than the EU27 average and, generally, than rates among Western countries.^29^

A case study is given to us when looking into breast cancer rate and mortality, which appear to be greater for CEE.^30^ In countries such as Poland and Romania, early detection programs seemed to be less effective and more recently developed programs have not been implemented yet.^29^ Some CEE countries are also seen to have an average longer time for diagnosis: In Poland, the average time for diagnosis on breast cancer takes 9.5 weeks and could even reach 38 weeks.^29^ Another issue is constituted by lower availability of novel cancer treatment medications in Eastern countries,^29^ which constitute a barrier toward equal access to care in Europe. Moreover, although for all stages of breast cancer combined, Western European countries have a 5-year survival rate of 80%; this rate is still lower for CEE countries.^31^ Finally, between 1989 and 2006, there was a median reduction in breast cancer mortality of 19%, but the range of this reduction was unfavorable toward Eastern European countries, for example, Iceland showed a reduction of 45%, whereas Romania actually showed an increase of 17%.^32^ In the same study, it appeared that in CEE mortality not only did not decline but in some cases, like Romania, actually increased.^32^

## Health Literacy

Health literacy can be a crucial driver to combat inequalities. There is increasing effort to promote shared decision making.^33^ That process is only possible if patients and their families can understand all the information regarding the whole treatment pathway. When it comes to evaluating treatment options, patients should be fully informed about the potential benefits and harms of treatment plans. Moreover, as health care progressively becomes more digitalized, digital literacy grows in importance too.

In cancer care, having limited nondigital and digital health literacy are barriers that hinder engagement with digital technology, especially among older people, younger individuals, people with lower socioeconomic status, and geographically disadvantaged groups.^34^ These groups generally have less access to technologization and information about cancer care, which has resulting impact in limiting opportunity for navigating cancer care and, potentially, engaging with preventive care. If systematically the same social groups are not equipped with the needed skills to understand information, this can be seen as a systemic issue that perpetuates specific discrimination. Improving health literacy is strongly linked to the fight against inequalities in cancer care, as it can empower individuals and make them more conscious of, and more engaged with, the health system.

## When, if Not Now?

The EU has never had a moment of opportunity on tackling cancer together like the present moment. The political will and support from key EU institutions is manifest. The initiatives promised by EBCP and the EU Cancer Research Mission demonstrate impact from the wide range of targeted awareness raising and debate stimulating activity of the Network can translate into active actions. Indeed, both EBCP and European Research Mission on Cancer have been authored with a prominent attention to combatting cancer inequalities in mind (Table 1).

**TABLE 1:**
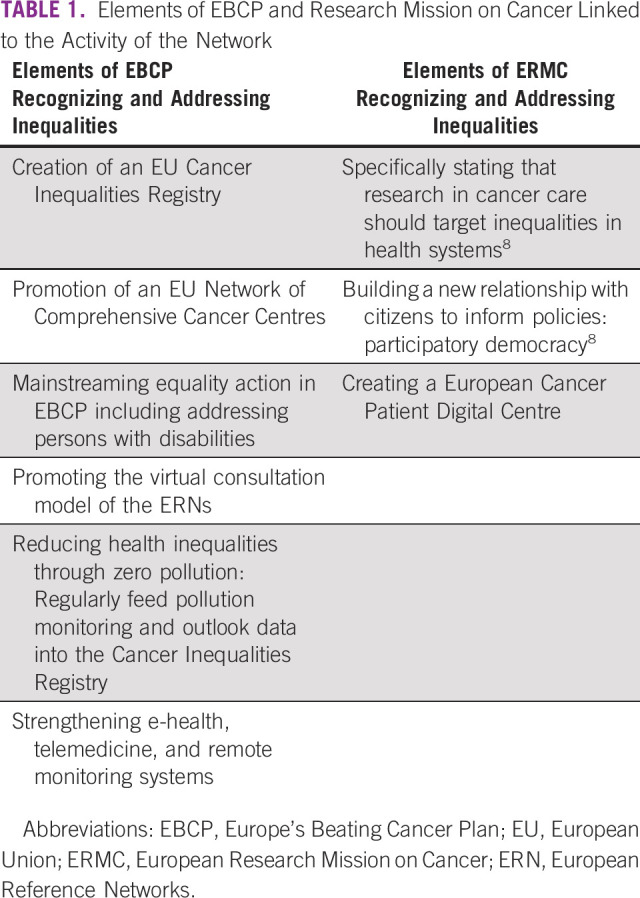
Elements of EBCP and Research Mission on Cancer Linked to the Activity of the Network

Given the impact of the advocacy on the implementation of new measures to address inequalities and to increase EU's awareness on the issue, ECO's Inequalities Network cannot but take its responsibility seriously to ensure that this momentum for implementation of practices for tackling inequalities is not missed.
